# Low 25-OH-vitamin D levels reflect hepatic dysfunction and are associated with mortality in patients with liver cirrhosis

**DOI:** 10.1007/s00508-016-1127-1

**Published:** 2016-11-25

**Authors:** Rafael Paternostro, Doris Wagner, Thomas Reiberger, Mattias Mandorfer, Remy Schwarzer, Monika Ferlitsch, Michael Trauner, Markus Peck-Radosavljevic, Arnulf Ferlitsch

**Affiliations:** 1 Vienna Hepatic Hemodynamic Laboratory, Divison of Gastroenterology and Hepatology, Department of Internal Medicine III, Medical University of Vienna, Waehringer Guertel 18–20, 1090 Vienna, Austria; 2 Divison of Gastroenterology and Hepatology, Department of Internal Medicine III, Medical University of Vienna, Vienna, Austria; 3 Department of Surgery, Medical University of Graz, Graz, Austria

**Keywords:** Vitamin D, Cirrhosis, Mortality, Liver dysfunction, Portal hypertension

## Abstract

**Background and aims:**

Vitamin D deficiency is frequent in patients with cirrhosis. The aims of this study were to evaluate the relation of vitamin D status to portal hypertension, degree of liver dysfunction and survival.

**Methods:**

Patients with cirrhosis who have been tested for 25-OH-vitamin D levels were retrospectively included. Vitamin D deficiency was defined as 25-OH-vitamin D levels <10 ng/ml. Child–Pugh score, model for end-stage liver disease (MELD) and available hepatic venous pressure gradient (HVPG) were recorded. Mortality was documented during follow-up.

**Results:**

A total of 199 patients were included. Prevalence of vitamin D deficiency (<10 ng/ml) was 40% (79/199), with 14% in Child–Pugh stage A, 39% in Child–Pugh stage B and 47% in Child–Pugh stage C (*p* = 0.001). Vitamin D deficiency was more common in patients with clinically significant portal hypertension (CSPH, HVPG ≥ 10 mm Hg) than in patients without (43.5% vs. 24.4%, *p* = 0.025). Significantly more deaths were observed in patients with vitamin D deficiency (32.9%, 26/79 vs. 13.3%, 16/120; *p* = 0.001). COX regression found presence of hepatocellular carcinoma (*p* < 0.001; HR: 5.763 95%CI:2.183–15.213), presence of CSPH (*p* = 0.026; HR: 5.487 95%CI: 1.226–24.55) and Child–Pugh stage C (*p* = 0.003; HR:5.429 95%CI: 1.771–16.638) as independent risk factors for mortality. Furthermore we could show a tendency towards group vitamin D deficiency being an independent risk factor (*p* = 0.060; HR: 1.86 95%CI:0.974–3.552).

**Conclusions:**

Vitamin D levels progressively decrease in more advanced Child stages and in patients with increasing HVPG. Vitamin D deficiency might be a valuable predictor of mortality in cirrhosis.

## Introduction

Liver cirrhosis is the cause of around 170,000 deaths per year in Europe, whereas liver cancer is responsible for around 47,000 deaths per year in the EU [1], while a recent report suggests that even this might be an underestimation [2]. There are several risk factors for developing complications of cirrhosis such as ascites, hepatic encephalopathy, and GI bleeding. Portal hypertension is the triggering factor for those complications most of the time since it decreases liver function and therefore encourages complications to begin [3].

The liver plays a crucial role in the biosynthesis of active vitamin-D3 (i. e., calcitriol or 1,25-OH vitamin-D3) via hydroxylation to 25-OH-vitamin-D3. Although the final hydroxylation step to produce 1,25-OH-vitamin-D3 is done in the kidney, 25-OH-vitamin-D3 (= VIT-D)—as synthesized by the liver—is the most commonly used biomarker to measure vitamin-D status in patients, given its half-life [4]. Other than that 25-OH-vitamin-D3 is also a key modulator of bone growth and remodeling [5].

In a large Austrian multicenter study, up to 42% of patients were found with VIT-D levels <20 ng/ml [6]. Various studies have shown that VIT-D deficiency (defined as VIT-D < 20 ng/ml in those studies) has an even higher prevalence in patients with CLD (chronic liver disease) ranging from 64 to 92% [7–9]. Especially in patients with cirrhosis VIT-D deficiency seems to be seen with a significant higher prevalence than in those without shown in a study performed by Fisher et al. [8] where 86% of cirrhotic patients had VIT-D deficiency compared to only 49% without cirrhosis. Also an inverse correlation between VIT-D status and severity of the liver disease was found by showing that patients with Child–Pugh score C had significantly lower mean VIT-D levels that those patients found with Child–Pugh score A (9.0 ± 4.0 in CPS C vs. 18.3 ± 6.7 ng/ml in CPS A).

Four studies using different definitions for VIT-D deficiency have evaluated the relationship between VIT-D levels of cirrhotic patients and mortality; significant correlations were found. However, conflicting results regarding the cut-off serum level for VIT-D as an independent risk factor for mortality were reported [10–13].

The aim of this study was to investigate how VIT-D influences mortality in a large cohort of patients and especially if it stands as an independent risk factor for mortality since the four studies performed reported conflicting results. Furthermore as portal hypertension is the main risk factor in the course of patients with cirrhosis we investigated the HVPG and its correlation with VIT-D levels to find whether VIT-D could be used as a noninvasive tool to predict portal pressure.

## Materials and methods

### Patients

In this retrospective multicenter study 199 patients diagnosed with liver cirrhosis that had VIT-D level available were included. A total of 100 consecutive patients were included from the Medical University of Graz and 99 consecutive patients were included from the Medical University of Vienna. Inclusion criteria were the following: available VIT-D level at time of study inclusion, available Child–Pugh score, available MELD score [14], no VIT-D supplementation of any kind at study inclusion and available follow-up for mortality. VIT-D levels were measured with routine clinical analyses in all patients during outpatient visits or administration to the ward. All patients have been diagnosed with cirrhosis based on either clinical, radiological parameters or on liver histology. Supplementation of VIT-D after study inclusion was left to the discretion of the physician following the patient in routine care and was not part of the study. Patients that underwent liver transplantation were censored at the day of transplantation. Patients selected for HVPG were cirrhotic patients routinely scheduled for response guided NSBB therapy or risk assessment prior to liver resection.

### Measurement of 25-OH-vitamin D

Blood used for analysing 25-OH-vitamin-D3 was drawn during routine blood-analysis when patients visited the outpatient clinic or at the ward. Chemiluminescence Immunoassay was used for exact measurement of 25-OH-vitamin-D3 (DiaSorin^TM^, Liaision XL, Saluggia, Italy). Reference range was 30–100 ng/ml. Vitamin D deficiency is defined as 25-OH-vitamin-D3 levels below 20 ng/ml [15–18] although several studies used different definitions of deficiency and took levels <10 ng/ml as deficient [12, 19]. Insufficiency is defined as 20–30 ng/ml [15–18] but once more several studies already used >20 ng/ml as the cut-off for normal VIT-D status in patients with CLD [10, 20, 21].

### HVPG measurement

The right internal jugular vein was accessed under ultrasound guidance and local anaesthesia with Seldinger technique using a catheter introducer set (8.5 F, Arrow International, Reading, PA, USA). Then a balloon catheter (Pejcl Medicintechnik, Austria)[22] was chosen to cannulate the liver vein via the transjugular access as described previously [23]. CSPH was defined as ≥10 mm Hg [24].

### Transient elastography

Transient elastography (Fibroscan, Echosense, France) correlates well with grade of fibrosis/cirrhosis [25]. It was performed in a supine position with the right arm resting behind the head of the patient before measurement of HVPG. At least 10 values were performed for each patient and the median value was then taken into account. Only values with an IQR/M < 30% were taken in account for statistical analysis [26].

### Statistical analysis

Continuous variables were reported as mean ± standard deviation (SD) or median (95% interquartile range [IQR]), and categorical variables were reported as number (*n*) of patients with the certain characteristic (proportion of patients with the certain characteristics [%]). Student t test was used for group comparisons of normally distributed data, and the Mann–Whitney U test where data was not normally distributed. Pearson’s Chi-Square test or Fishers exact test was performed to conduct group comparisons for categorical data. The impact of VIT-D on mortality incidence and transplant-free survival was analyzed using semi-parametric proportional hazard COX models. To minimize problems of multilinearity the following covariates were chosen: age, HCC yes/no, CSPH, CPS, status D‑DEF/D-NON-DEF. Child–Pugh score was chosen over MELD since it covers more aspects of cirrhosis (ascites, hepatic encephalopathy, albumin and prothrombin-time). Patients entered the model on the day when blood was drawn for analysing VIT-D levels and were followed until either (I) death (II) liver transplantation or (III) lost of follow-up. Patients who received liver transplantation were censored at the day of surgery. A multiple linear regression model was used to find independent covariates that influence absolute VIT-D levels (independent variable: absolute VIT-D levels). A binary logistic model was conducted to find independent risk factors of being found with VIT-D levels below 10 ng/ml (independent variable: D‑DEF yes/no). Kaplan–Meier curves are shown for comparison of survival time in patients. Log-rank test was conducted to find difference in mean survival times. Two sided *p* values <0.05 were considered as statistically significant. The IBM SPSS 22.0 statistic software (SPSS Inc., Armonk, NY, USA) was used for all statistical analysis.

## Results

In total, 199 patients were included in the study. For main patient characteristics see Table 1. We classified patients into two groups for all statistical analyses: VIT-D < 10 ng/ml = D‑DEF and VIT-D > 10 ng/ml = D‑NON-DEF.

**Table 1:** Patients characteristics with and without vitamin D deficiency

Patients characteristics	All patients (*n* = 199)	Correlation (absolute VIT-D [ng/ml]) *P* value	VIT-D ≤ 10 ng/ml (*n* = 79)	VIT-D > 10 ng/ml (*n* = 120)	*P* Value <10 ng/ml vs >10 ng/ml
25-Hydroxyvitamin D3 [ng/ml], median (95%CI)	11.98 (4–31.33)	–	6.41 (3.25–9.5)	17.4 (10.42–33)	<0.001
Age, median (95%CI)	57 (38–69)	0.037 (r = 0.148)	56 (38–72)	58 (38–69)	0.072
*Gender, n (%)*					
Male	147 (73.9%)	0.146 (r = −0.104)	57 (72.2%)	90 (75%)	0.742
Female	52 (26.1%)		22 (27.8%)	30 (25%)	–
*CPS, n (%)*					
A	57 (28.6%)	<0.001 (r = −0.235)	11 (13.9%)	46 (38.3%)	0.001
B	68 (34.2%)		31 (39.2%)	37 (30.8%)	
C	74 (37.2%)		37 (46.8%)	37 (30.8%)	
HVPG, mean ± SD	16 ± 6.3^a^	<0.001 (r = −0.360)	19 ± 6.13	14 ± 5.94	<0.001
*CSPH, n (%)*					
Yes	156 (79.1%)	0.001 (r = −0.235)	68 (87.2%)	88 (74%)	0.025
No	41 (20.9%)		10 (12.8%)	31 (26%)	
*HCC, n (%)*					
Yes	27 (13.6%)	0.091 (r = −0.120)	10 (12.7%)	17 (14.2%)	0.835
No	172 (86.4%)		69 (87.3%)	103 (85.8%)	
MELD, median (95%CI)	12 (6.43–24)	0.002 (r = −0.223)	13 (6.9–26.6)	11 (6.4–20)	0.003
Bilirubin [mg/dl], median (95%CI)	1.85 (0.48–15.17)	0.004 (r = −0.203)	2.2 (0.48–23.13)	1.54 (0.5–5.6)	0.007
Albumin [g/l] mean ± SD	33.9 (±6.2)	<0.001 (r = 0.274)	31.8 (±5.74)	35.34 (±6.15)	<0.001
Prothrombin, time [%], median (95%CI)	62 (31–106)	<0.001 (r = 0.305)	56 (25–41)	67 (36.8–107.1)	0.001
Transient elastography [kPa], median (95%CI)	44 (12.6–75)^b^	0.003 (r = −0.425)	69.5 (11.66–75)	32.5 (12.33–75)	0.003
Days of follow-up, median (95%CI)	419 (22–1048)	0.189 (r = 0.093)	294 (11–1027)	446 (22–1049)	0.109
Death, *n* (%)	42 (21.1%)	0.002 (r = −0.216)	26 (32.9%)	16 (13.3%)	0.001

^a^Available in 197 patients

^b^Available in 48 patients

### Vitamin D and cirrhosis

**Fig. 1:**
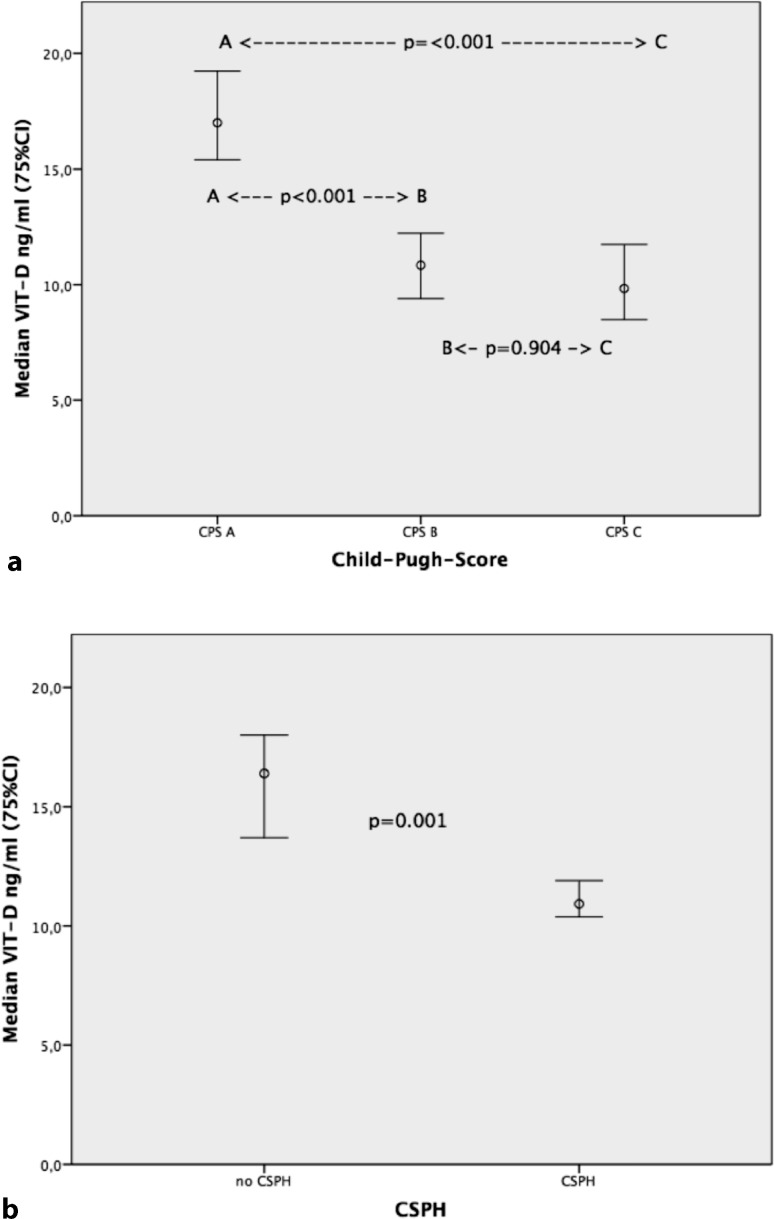
**a** Median 25-OH-vitamin-D3 (*VIT-D*) levels over groups of Child–Pugh score stages. **b** Median VIT-D levels in patients with and without clinical significant portal hypertension (*CSPH*)

Of patients, 28.6% were found in CPS A, 34.2% in CPS B and 37.2% in CPS C. Significant correlation with absolute VIT-D levels was found (*p* < 0.001, r = −0.251). We also compared median values in all three groups and found significant difference (CPS A: 17 ng/ml, range 11.1–26, CPS B: 10.8 ng/ml, range 7.1–15.5, CPS C: 9.8 ng/ml, range 6.1–18.2; A vs. B *p* < 0.001; A vs. C *p* < 0.001; B vs. C *p* = 0.904, see Fig. 1a). Also absolute VIT-D levels significantly correlated with MELD score (*p* = 0.002, r = −0.223). When separated into groups MELD score >10 vs ≤10 significant differences in median VIT-D levels were found (11.2 ng/ml vs. 14.6 ng/ml; *p* = 0.013). Significant correlation between VIT-D values and prothrombin time values was found (*p* < 0.001, r = 0.305). Univariate binary logistic regression (independent variable: prothrombin time <60%/prothrombin time >60%, covariate: status D‑DEF/D-NON-DEF) found being in 10-DEF as a risk factor for group “prothrombin time <60%” (*p* = 0.002; OR: 2.528 95%CI: 1.404–4.552). Also Mann–Whitney U test found significant differences in median VIT-D values between the two subgroups (“prothrombin time >60%” 14.4 ng/ml vs. “prothrombin time <60%” 9.2 ng/ml; *p* < 0.001).

### Vitamin D and portal pressure

In total, 78.4% of all patients were found with clinical significant portal hypertension (CSPH). We found significant correlations of absolute VIT-D with HVPG values (*p* < 0.001, r = −0.360) as well as presence of CSPH (*p* = 0.001, r = −0.235). Also significant differences in median VIT-D levels were found between the CSPH vs. no-CSPH group (10.9 ng/ml vs. 16.4 ng/ml, *p* = 0.001, see Fig. 1b). When comparing groups D‑DEF/D-NON-DEF significantly higher distribution of CSPH within the D‑DEF group could be found (87% of D‑DEF had CSPH vs only 74% of D‑NON-DEF patients; *p* = 0.025). ROC analysis found an AUC of 0.667 (*p* = 0.001) for the prediction of CSPH using VIT-D.

### Vitamin D and mortality

**Fig. 2:**
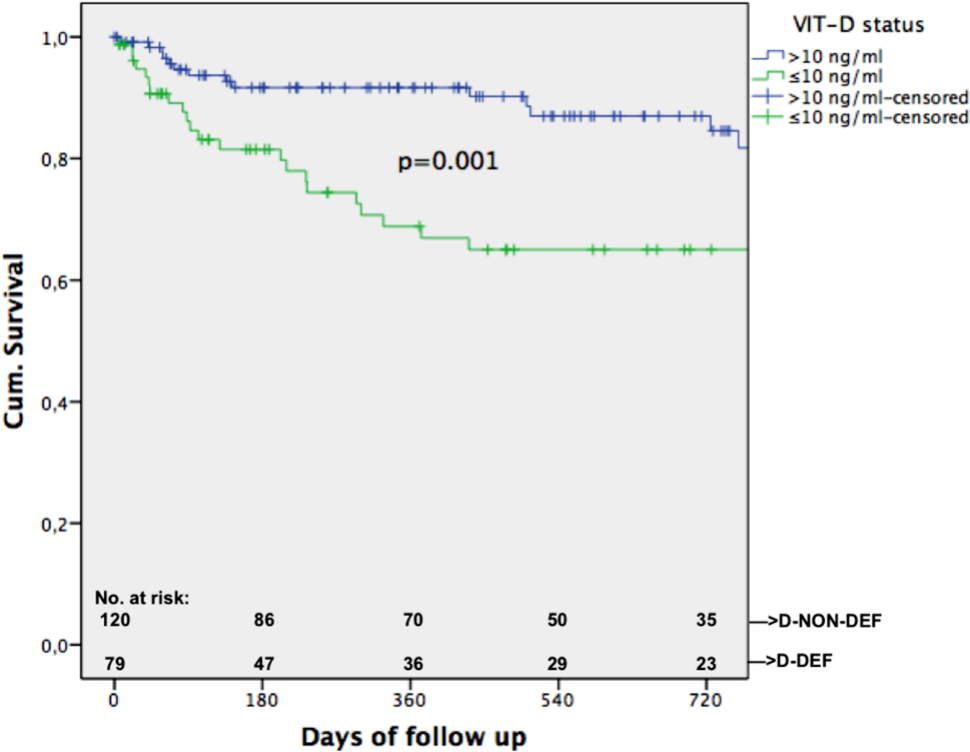
Kaplan–Meier curve shown for all patients separated in groups with vitamin-D-deficiency (D-DEF) and without (*D-NON-DEF*)

**Fig. 3:**
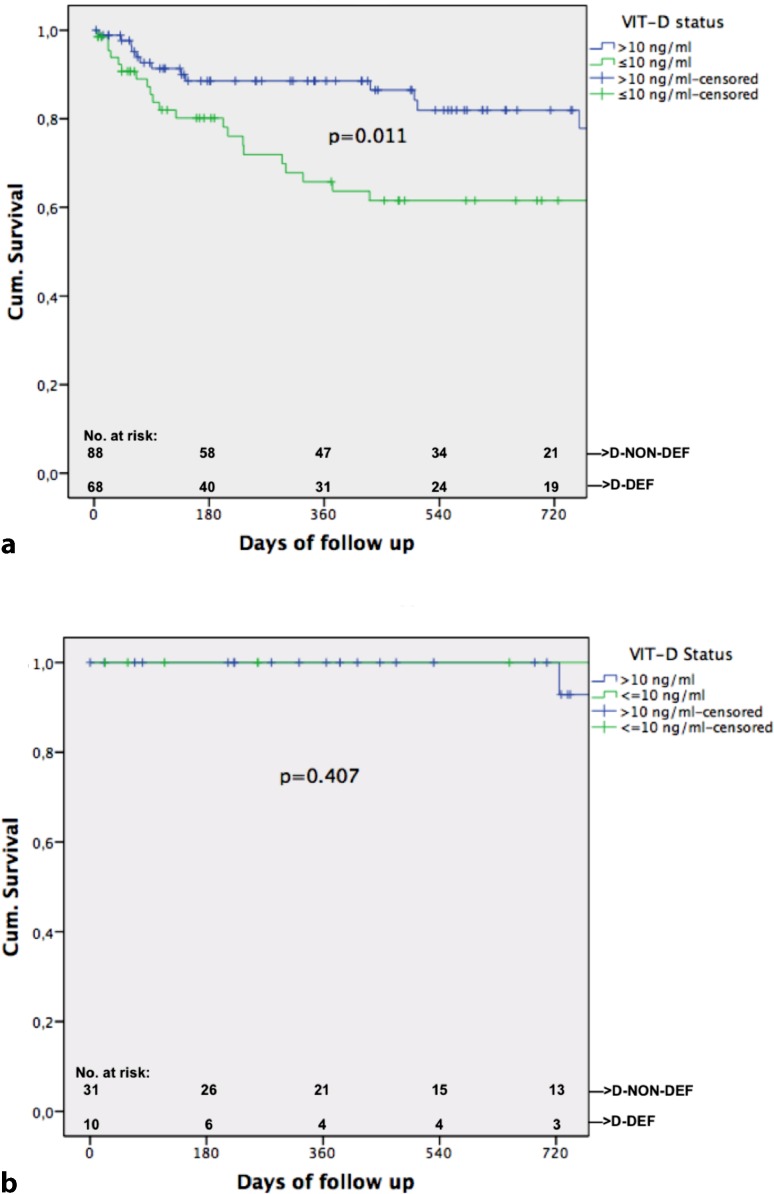
Kaplan–Meier curve shown for patients with (**a**) and without (**b**) clinical significant portal hypertension (*CSPH*) separated in groups with vitamin-D deficiency (D-DEF) and without (*D-NON-DEF*)

**Fig. 4:**
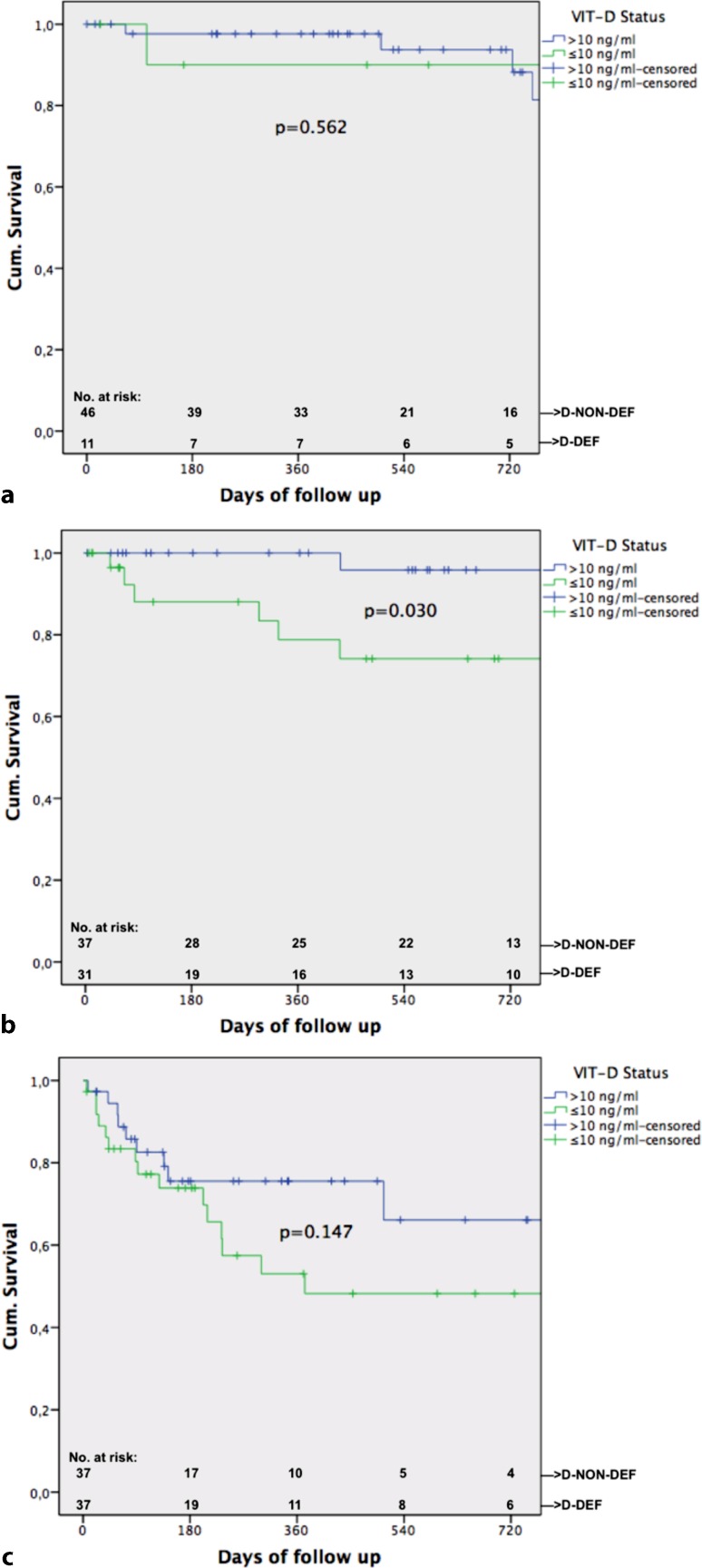
Kaplan–Meier curve for each Child–Pugh score (*CPS*) stage separated in groups with vitamin-D deficiency (*D-DEF*) and without (*D-NON-DEF*)

In all, 42/199 (21.1%) patients died during follow-up. Univariate correlation analysis found the following parameters significantly associated with death: albumin (*p* = 0.002, r = −0.223), MELD (*p* = 0.006; r = 0.194), HVPG (*p* = 0.003, r = 0.210), CPS (*p* = 0.001, r = 0.238) and absolute VIT-D (*p* = 0.002, r = −0.216). Significantly more patients died in group D‑DEF 26/79 (= 32.9%) than in group D‑NON-DEF 16/120 (= 13.3%) (*p* = 0.001). Significant difference in median VIT-D levels in groups “death” vs. “no death” was found (*p* = 0.002; 7.95 ng/ml [4–32.3] vs. 12.8 ng/ml [4.1–31.3]). COX regression (covariates: age, HCC yes/no, CSPH, CPS, status D‑DEF/D-NON-DEF) found presence of HCC (*p* < 0.001; HR: 5.763 95%CI: 2.183–15.213), presence of CSPH (*p* = 0.026; HR: 5.487 95%CI: 1.226–24.55) and CPS C (*p* = 0.003; HR:5.429 95%CI: 1.771–16.638) as independent risk factors for mortality. A trend towards being in group D‑DEF could be seen (*p* = 0.060; HR: 1.86 95%CI: 0.974–3.552). Kaplan–Meier curve found significant a difference in survival in groups D‑DEF/D-NON DEF (*p* = 0.001, see Fig. 2). Furthermore we analyzed subgroups and the influence of D‑DEF status in their survival (see Figs. 3 and 4). ROC analysis determined an AUC of 0.653 (*p* = 0.002) for the predicting value of absolute VIT-D levels for death.

### Vitamin D and transient elastography

A total of 48 patients had valid (IQR < 30% of total kPa) TE tracings. Significant different values were found in groups D‑DEF/D-NON-DEF (Table 1). Correlation analysis found significant correlation between absolute VIT-D values and stiffness (*p* = 0.003, r = −0.425).

### Multivariate analysis

Linear regression (independent variable: absolute VIT-D values; covariates *model 1:* age, HCC, CSPH, MELD; *model 2:* age, HCC, CSPH, CPS) found CSPH as an independent risk factor for low absolute VIT-D levels in both models(*model 1: p* = 0.017, *model 2: p* = 0.035). Multivariate analysis found that patients found with CPS B (*p* = 0.004; OR: 3.317 95%CI: 1.466–7.504) and CPS C (*p* = 0.001; OR:4.091 95%CI:1.836–9.117) are at higher risk for being found with VIT-D levels below <10 ng/ml.

## Discussion

Our study investigated the correlation of Vitamin D in portal hypertension (documented by HVPG) and its predictive value in patients with liver cirrhosis in a large cohort of patients. We could show up to 94% of patients with liver cirrhosis suffer from VIT-D deficiency when taking >30 ng/ml as normal level, whereas 76.4% where found deficient when taking <20 ng/ml as the cut-off and 40% with levels <10 ng/ml. This results are in line with the literature when 20 ng/ml was taken as a cut-off; Stokes et al. [11] reported that 86% of cirrhotic patients were deficient and a recent study found 68.9% of patients with VIT-D < 10 ng/ml [13]. Another study performed in Austria by Putz-Bankuti et al. [10] found 71% deficient (<20 ng/ml) patients so the real number might be somewhere around 80%. Those results also fit the literature for patients with chronic liver disease (CLD) where Lange et al. [21] found 66% of chronic HCV patients in a deficient state. Therefore it seems that presence of cirrhosis severely increases prevalence of VIT-D deficiency. Regarding correlation between grade of liver dysfunction and VIT-D we found significant differences between CPS A vs. B and A vs. C, and could therefore enhance the results found in other studies [10, 13]. Regarding MELD scores we could confirm the results found in Putz Bankuti et al. [10] and Finkelmeier et al. [13] that absolute VIT-D significantly correlated with MELD score.

Regarding HVPG we confirm the results found by Trepo et al. [12] published in 2013 that higher HVPG values are associated with low VIT-D. Furthermore we found CSPH as an independent risk factor for low absolute VIT-D in multivariate analysis. Also median VIT-D levels were significantly lower in the CSPH group (Fig. 1b) and significantly more patients with D‑DEF status were found in the CSPH group (Table 1). We could for the first time show that VIT-D significantly inversely correlates with absolute transient elastography values. Significant differences in median TE values could be found in groups D‑DEF/D-NON-DEF (Table 1). This confirms the results by Trepo et al. [12] where a significant association between absolute VIT-D steatosis and fibrosis was found. This underlines the possible potential value of VIT-D as a non-invasive parameter for staging cirrhosis.

Regarding mortality significantly lower median VIT-D was found in patients who died during follow-up (*p* = 0.002). We also found presence of HCC, presence of CSPH and CPS C as independent risk factors for mortality. Although D‑DEF marginally missed significance we could show a trend towards vitamin D status as an independent risk factor for mortality (Table 2). Therefore our results are in line with the results found in previous studies that VIT-D is a valuable predictor for survival [10–13, 27]. We could also show that D‑DEF seems to be a good cut-off for mortality given our Kaplan–Meier curve for all patients (Fig. 2). For the first time we could also show that VITD seems to be a significant factor regarding survival when analyzing subgroup of patients with CSPH and patients with CPS B (Figs. 3 and 4). Interestingly in patients with no CSPH and also in those with CPS A D-DEF was not a significant factor associated with survival of those patients; hence this could be due to their still rather intact liver. CPS C also did not reach significance in this subgroup analysis, but our graph fairly shows that those patients seem to decompensate very shortly after inclusion due to their severe liver damage. Therefore it seems D‑DEF is not able to predict their outcome given their end-stage disease status.

**Table 2:** Multivariate stepwise backwards COX regression analysis regarding factors independently associated with mortality

Co-Variates	*p*-value	Hazard ratio	95%CI
Presence of HCC	<0.001	5.763	2.183–15.213
Presence of CSPH	0.026	5.487	1.226–24.55
CPS C	0.003	5.429	1.771–16.638
Status D‑DEF	0.060	1.86	0.974–3.552

Co-variates: age, HCC yes/no, CSPH, CPS, status D‑DEF/D-NON-DEF

*VIT-D* 25-OH-vitamin-D3, *CPS* Child–Pugh score , *HVPG* hepatic venous pressure gradient, *CSPH* clinical significant portal hypertension, *HCC* hepatocellular carcinoma, *MELD* model for end-stage liver disease

Stokes et al. very well described the function of VIT-D in liver disease in their review published in 2012 [4]. Reduced exogenous exposure of patients to VIT-D sources, intestinal malabsorption of dietary VIT-D3, reduced endogenous production of VIT-D binding protein and albumin, impaired hepatic hydroxylation of 1,25-OH-vitamin D3 to 25-OH-vitamin D3 and increased catabolic removal of 25-OH-vitamin D3 are described as responsible mechanisms for the VIT-D deficiency in cirrhosis [4]. An association between liver-related complications and low VIT-D levels have been shown by Trepo et al. [12], Wong GL et al. [28] and most recently by Finkelmeier et al. [13]. Trepo et al. described a significantly higher rate of PHT complications (such as ascites, HE or HRS) when patients were found with VIT-D levels <10 ng/ml; hence we chose our group D‑DEF (<10 ng/ml) to be able to properly compare results. Wong et al. [28] found that in a large prospective HBV cohort (*n* = 426, 11% found with cirrhosis, 89% with CLD) patients that developed clinical events had significantly lower VIT-D levels than those who did not. Finkelmeier et al. [13] on the other hand found that patients with diagnosed SBP had significant lower VIT-D levels than those without. Hence the previous results regarding mortality and decompensation are therefore in line with the results found in our study. In a recently published paper by Lai JC et al. [29] the relationship between VIT-D levels, albumin and vitamin-D binding protein (DBP) was shown. They found that cirrhotics with synthetic dysfunction (= albumin < 3.5 g/dl) tend to have lower total and free VIT-D as well as DBP levels but higher percentage of free VIT-D. They stated that “total VIT-D is not an accurate marker of true Vitamin D status” and furthermore proposed that “supplementation may not be an adequate therapy for bone disease in cirrhosis”. Regarding our study we therefore propose that patients found with VIT-D under 10 ng/ml should be screened and evaluated even more tightly given the results by Lai JC et al. Hence even though their VIT-D status might not be accurate we nevertheless found increased risk for adverse events in those lower than 10 ng/ml. Furthermore we investigated VIT-D as a noninvasive marker for grade of disease and prediction of mortality and did not aim to evaluate its role in cirrhotic bone disease.

In conclusion there is a strong trend towards VIT-D levels predicting mortality in patients with cirrhosis and a 10 ng/ml cut-off seems to discriminate patients at higher risk for mortality. Moreover VIT-D seems to be an accurate marker of reflecting liver dysfunction and is a good synthesis-related parameter. Although we found significant results regarding the association of VIT-D and transient elastography, given the small number of patients, further prospective studies are needed in that direction to prove the possible value of a combined noninvasive screening marker. Prediction of CSPH through VIT-D cannot be made.

## Abbreviations

VIT-D: 25-OH-vitamin D3
HVPG: Hepatic venous pressure gradient
CPS: Child–Pugh score
CLD: Chronic liver disease
CSPH: Clinical significant portal hypertension
D-DEF: VIT-D levels <10 ng/ml
D-NON-DEF: VIT-D levels >10 ng/ml
HCC: Hepatocellular carcinoma
kPa: Kilo Pascal
TE: Transient elastography
HCV: Hepatitis C virus
NAFLD: Nonalcoholic fatty liver disease
PHT: Portal hypertension
HE: Hepatic encephalopathy
HRS: Hepatorenal syndrome
HBV: Hepatitis B virus
SBP: Spontaneous bacterial peritonitis
DBP: D-binding protein

## References

[1] Blachier M, Leleu H, Peck-Radosavljevic M (2013). The burden of liver disease in Europe: a review of available epidemiological data. J. Hepatol..

[2] Asrani SK, Larson JJ, Yawn B (2013). Underestimation of liver-related mortality in the United States. Gastroenterology.

[3] D’Amico G, Garcia-Tsao G, Pagliaro L (2006). Natural history and prognostic indicators of survival in cirrhosis: a systematic review of 118 studies. J. Hepatol..

[4] Stokes CS, Volmer DA, Grunhage F (2013). Vitamin D in chronic liver disease. Liver Int.

[5] Plum LA, DeLuca HF (2010). Vitamin D, disease and therapeutic opportunities. Nat Rev Drug Discov.

[6] Muschitz C, Kocijan R, Stutz V (2015). Vitamin D levels and comorbidities in ambulatory and hospitalized patients in Austria. Wien. Klin. Wochenschr..

[7] Arteh J, Narra S, Nair S (2010). Prevalence of vitamin D deficiency in chronic liver disease. Dig. Dis. Sci..

[8] Fisher L, Fisher A (2007). Vitamin D and parathyroid hormone in outpatients with noncholestatic chronic liver disease. Clin Gastroenterol Hepatol.

[9] Chen CC, Wang SS, Jeng FS (1996). Metabolic bone disease of liver cirrhosis: is it parallel to the clinical severity of cirrhosis?. J. Gastroenterol. Hepatol..

[10] Putz-Bankuti C, Pilz S, Stojakovic T (2012). Association of 25-hydroxyvitamin D levels with liver dysfunction and mortality in chronic liver disease. Liver Int.

[11] Stokes CS, Krawczyk M, Reichel C (2014). Vitamin D deficiency is associated with mortality in patients with advanced liver cirrhosis. Eur. J. Clin. Invest..

[12] Trepo E, Ouziel R, Pradat P (2013). Marked 25-hydroxyvitamin D deficiency is associated with poor prognosis in patients with alcoholic liver disease. J. Hepatol..

[13] Finkelmeier F, Kronenberger B, Zeuzem S (2015). Low 25-Hydroxyvitamin D Levels Are Associated with Infections and Mortality in Patients with Cirrhosis. PLOS ONE.

[14] Kamath PS, Kim WR (2007). The model for end-stage liver disease (MELD). Hepatology (Baltimore Md).

[15] Holick MF (2006). High prevalence of vitamin D inadequacy and implications for health. Mayo Clin. Proc..

[16] Bischoff-Ferrari HA, Giovannucci E, Willett WC (2006). Estimation of optimal serum concentrations of 25-hydroxyvitamin D for multiple health outcomes. Am. J. Clin. Nutr..

[17] Malabanan A, Veronikis IE, Holick MF (1998). Redefining vitamin D insufficiency. Lancet.

[18] Dawson-Hughes B, Heaney RP, Holick MF (2005). Estimates of optimal vitamin D status. Osteoporos Int.

[19] Malham M, Jørgensen Sø P, Ott P (2011). Vitamin D deficiency in cirrhosis relates to liver dysfunction rather than aetiology. World J. Gastroenterol..

[20] Bitetto D, Fattovich G, Fabris C (2011). Complementary role of vitamin D deficiency and the interleukin-28B rs12979860 C/T polymorphism in predicting antiviral response in chronic hepatitis C. Hepatology (Baltimore Md).

[21] Lange CM, Bojunga J, Ramos-Lopez E (2011). Vitamin D deficiency and a CYP27B1-1260 promoter polymorphism are associated with chronic hepatitis C and poor response to interferon-alfa based therapy. J. Hepatol..

[22] Ferlitsch A, Bota S, Paternostro R (2015). Evaluation of a new balloon occlusion catheter specifically designed for measurement of hepatic venous pressure gradient. Liver Int.

[23] Ferlitsch M, Reiberger T, Hoke M (2012). Von Willebrand factor as new noninvasive predictor of portal hypertension, decompensation and mortality in patients with liver cirrhosis. Hepatology (Baltimore Md).

[24] Peck-Radosavljevic M, Angermayr B, Datz C (2013). Austrian consensus on the definition and treatment of portal hypertension and its complications (Billroth II). Wien. Klin. Wochenschr..

[25] Reiberger T, Ferlitsch A, Payer BA (2012). Noninvasive screening for liver fibrosis and portal hypertension by transient elastography – a large single center experience. Wien. Klin. Wochenschr..

[26] Schwabl P, Bota S, Salzl P (2015). New reliability criteria for transient elastography increase the number of accurate measurements for screening of cirrhosis and portal hypertension. Liver Int.

[27] Wang JB, Abnet CC, Chen W (2013). Association between serum 25(OH) vitamin D, incident liver cancer and chronic liver disease mortality in the linxian nutrition intervention trials: a nested case-control study. Br J Cancer.

[28] Wong GL, Chan HL, Chan HY (2015). Adverse effects of vitamin d deficiency on outcomes of patients with chronic hepatitis B. Clin Gastroenterol Hepatol.

[29] Lai JC, Bikle DD, Lizaola B (2015). Total 25(OH) vitamin D, free 25(OH) vitamin D and markers of bone turnover in cirrhotics with and without synthetic dysfunction. Liver Int.

